# Primary small cell type of non-Hodgkin lymphoma of the colon: a case report

**DOI:** 10.1186/s13256-020-02500-y

**Published:** 2020-09-19

**Authors:** Eyal Meir, Chovav Handler, Uri Kaplan, Doron Kopelman, Ossama A. Hatoum

**Affiliations:** 1 Department of Surgery B, Emek Medical Center, Afula, Israel; 2 Rapaport Faculty of Medicine, Technion-Israel Institute of Technology, Haifa, Israel

**Keywords:** Case report, Colon, Lymphoma

## Abstract

**Introduction:**

Primary lymphoma of the colon is exceedingly rare and comprises 0.2–1% of all colon tumors. The most common subtype of lymphoma in the colon is non-Hodgkin lymphoma. Symptoms are often nonspecific, and treatment varies between chemotherapy alone and a combination of surgery and chemotherapy.

**Case presentation:**

We describe a case of a Ashkenazi Jew patient who presented in the typical way that carcinoma of the colon might present but turned out to have a very rare type of tumor in both its histology and its location.

**Conclusion:**

There was apparent discordance between the relative bulkiness and gross appearance of the tumor with the unrevealing result of the biopsies, demanding a high level of suspicion as to the actual presence and possible type of such a tumor in the future.

## Introduction

Primary lymphoma of the colon is exceedingly rare and comprises 0.2–1% of all colon tumors [1–4]. The most common subtype of lymphoma in the colon is non-Hodgkin lymphoma (NHL) [5]. Though the most common site for secondary spread of lymphoma is the gastrointestinal (GI) tract, primary lymphoma of the GI tract accounts for only 10–15% of all lymphomas. The most common GI location for primary lymphoma is the stomach 25-50%, followed by the small intestine (20–30%). The colon and rectum account for the remaining 10–20% [6, 7]. Symptoms are often nonspecific, and treatment varies between chemotherapy alone and a combination of surgery and chemotherapy [8].

## Case presentation

**Fig. 1:**
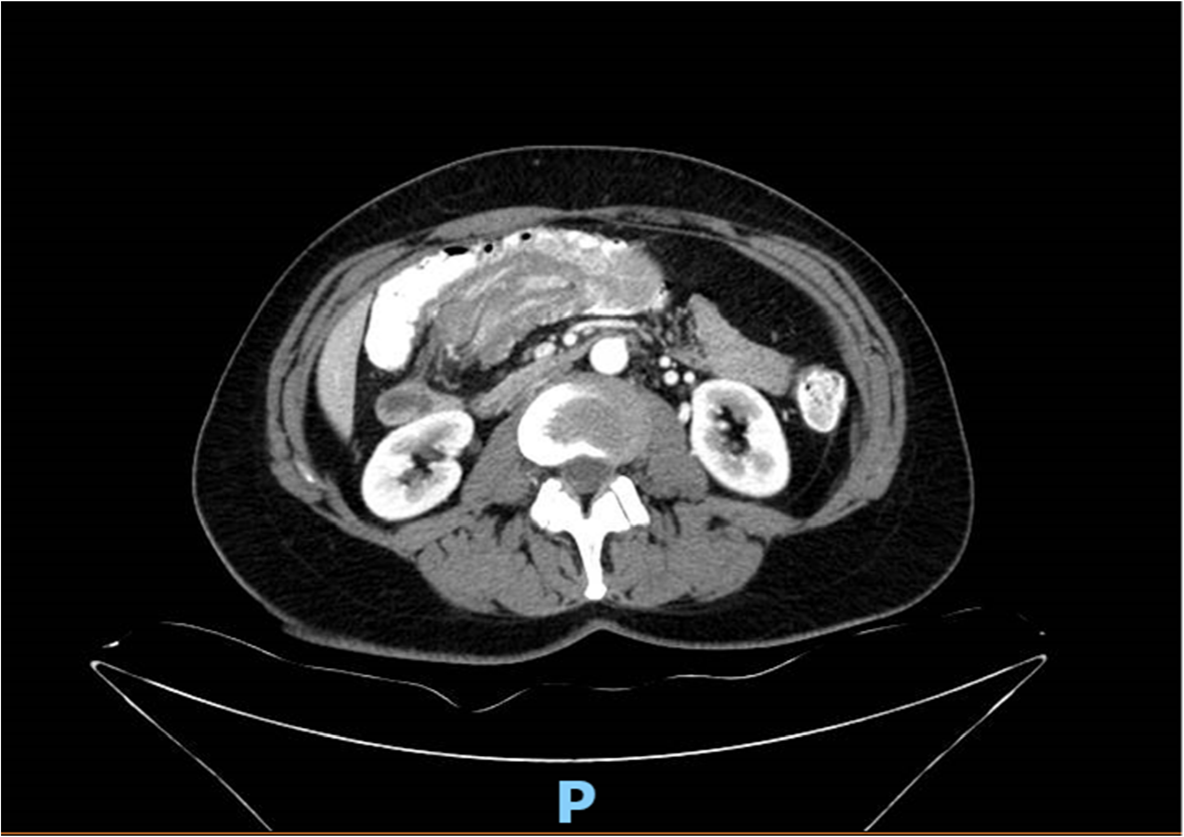
Initial presentation with a huge mass that occupies the whole colonic lumen and causes a colocolic intussusception (*arrow*)

**Fig. 2:**
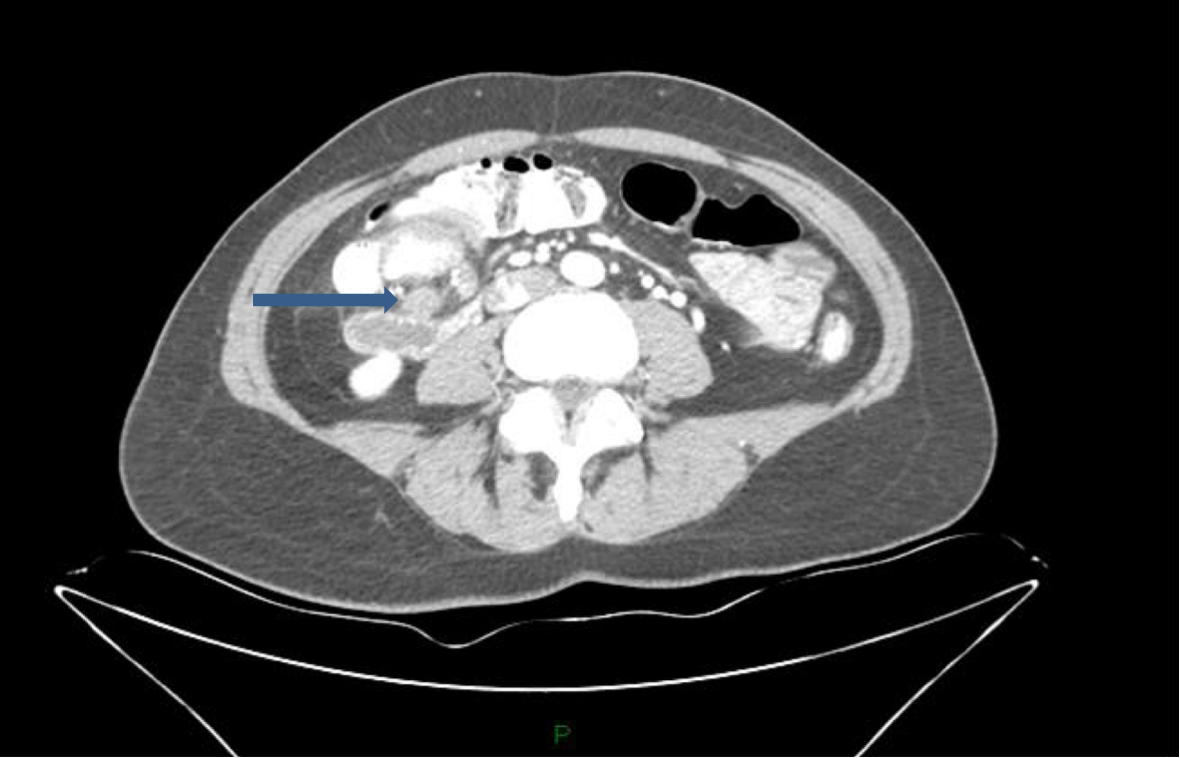
Considerable mesenteric lymphadenopathy (*arrow*)

A 57-year-old Ashkenazi Jew woman, who aside from iron deficiency anemia was relatively well, with no family or personal history of malignancy, was admitted to our department of general surgery for treatment of her transverse colon tumor. Four months prior, she had begun experiencing periumbilical abdominal pain hematochezia, and she had a 10-kg weight loss. Upon physical examination, no masses were palpated, and there were no other pathologic findings. She underwent a colonoscopy, which revealed a large mass that involved nearly the whole circumference of the colon and seemed to be adjacent to the cecum. Biopsies were taken that failed to demonstrate any colonic pathology. She proceeded to undergo computed tomography (CT) of the chest and abdomen that demonstrated a huge mass that occupied the whole colonic lumen and caused a colocolic intussusception (Fig. 1). Considerable mesenteric lymphadenopathy was seen with nodes up to 28 × 21 mm in diameter and was deemed to be evidence of positive tumoral lymph node involvement (Fig. 2). No inguinal, pelvic, retroperitoneal, or other lymphadenopathy was seen. Considering the gross endoscopic and CT findings, she was scheduled for surgery. A laparoscopic right extended hemicolectomy was performed, which was uncomplicated, and during which considerable mesocolic lymphadenopathy was seen and widely resected accordingly.

Pathology of the surgical specimen showed findings consistent with small B cell lymphoproliferative disorders (LPDs) with plasmacytoid differentiation. At this point, though primary lymphoma of the colon was considered in the differential diagnosis, the disease was thought to be part of systemic dissemination of lymphoma. The patient was referred to the hematology clinic for further investigation. A bone marrow biopsy was performed, and the result was normal. The investigation was complemented by positron emission tomography-CT, which showed no other focus of lymphoma. Also, the result of a test for Epstein-Barr virus infection as a possible predisposing factor for lymphoma was negative. These results support the diagnosis of a primary colonic NHL small B-cell LPD with plasmacytoid differentiation, an exceedingly rare disease with only two such reports in the current literature [2, 9].

## Discussion

B-cell lymphoma is the third most common tumor of the colon (following adenocarcinoma and carcinoid) but still comprises only 0.5% of all colonic tumors [10]. Despite its rarity, it should be noted that its frequency seems to be rising, especially in North America [11]. It is thought that primary NHL of the colon arises from mucosa-associated lymphatic tissue of the colon [5]. The subtype diffuse large B-cell lymphoma is the most common subtype, and the most common colonic site for this tumor is the cecum [5]. This is presumably due to the excess of lymphatic tissue in this region. The cecum is then followed by the right colon and the sigmoid colon. Symptoms described in the literature are varied and include abdominal pain, weight loss, hematochezia, abdominal mass, intussusception, intestinal obstruction, and even perforation and peritonitis [1, 3, 5, 10], and it is estimated that 20% of patients require emergent surgery [11]. This disease usually affects older people (the median age is older than 55 years) and mostly men [12]. Risk factors for colonic NHL are autoimmune diseases such as inflammatory bowel disease, celiac disease, and human immunodeficiency virus carrier status [13]. The treatment of colonic lymphoma includes a combination of surgery and chemotherapy [14] and depends on the staging of the disease. The lower stages of disease are treated with surgery and complemented by adjuvant polychemotherapy, whereas the more advanced stages are treated with multidrug chemotherapy (mostly CHOP [cyclophosphamide, doxorubicin, vincristine, prednisone]), or other combinations of chemotherapy and rituximab (R-CHOP [cyclophosphamide, doxorubicin, vincristine, prednisone, rituximab]) [10, 11, 15]. A recent review of the literature [10] that covered 20 publications regarding colonic lymphoma included only 2 cases of small cell lymphoma.

## Conclusions

In this case report, we describe a patient who presented in the typical way that carcinoma of the colon might present but turned out to have a very rare type of tumor in both its histology and its location. There was an apparent discordance between the relative bulkiness and gross appearance of the tumor and the unrevealing result of the biopsies. This type of case, in which the pathology is not conducive, demands that we maintain a high level of suspicion as to the actual presence and possible type of such a tumor. It demands that we remain familiar with the presentation and treatment options of the colonic lymphomas.

## Data Availability

Data sharing is not applicable to this article, because no datasets were generated or analyzed during the present study.
